# Glucose and glutamine metabolism in relation to mutational status in NSCLC histological subtypes

**DOI:** 10.1111/1759-7714.13226

**Published:** 2019-10-30

**Authors:** Tineke W.H. Meijer, Monika G. Looijen‐Salamon, Jasper Lok, Michel van den Heuvel, Bastiaan Tops, Johannes H.A.M. Kaanders, Paul N. Span, Johan Bussink

**Affiliations:** ^1^ Radiotherapy and OncoImmunology Laboratory, Department of Radiation Oncology Radboud University Medical Center Nijmegen The Netherlands; ^2^ Department of Pathology Radboud University Medical Center Nijmegen The Netherlands; ^3^ Department of Pulmonary Diseases Radboud University Medical Center Nijmegen The Netherlands

**Keywords:** Glutamine metabolism, glycolysis, mutational status, non‐small cell lung cancer

## Abstract

**Background:**

Both hypoxia and oncogenic mutations rewire tumor metabolism. In this study, glucose and glutamine metabolism‐related markers were examined in stage I ‐ resectable stage IIIA non‐small cell lung cancer (NSCLC). Furthermore, expression of metabolism‐related markers was correlated with mutational status to examine mutations associated with rewired tumor metabolism.

**Methods:**

Mutation analysis was performed for 97 tumors. Glucose and glutamine metabolism‐related marker expression was measured by immunofluorescent staining (protein) and qPCR (mRNA) (*n* = 81).

**Results:**

Glutamine metabolism‐related markers were significantly higher in adeno‐ than squamous cell NSCLCs. Glucose transporter 1 (GLUT1) protein expression was higher in solid compared to lepidic adenocarcinomas (*P* < 0.01). In adenocarcinomas, mRNA expression of glutamine transporter SLC1A5 correlated with tumor size (r(p) = 0.41, *P* = 0.005). Furthermore, SLC1A5 protein expression was significantly higher in adenocarcinomas with worse pTNM stage (r(s) = 0.39, *P* = 0.009). *EGFR*‐mutated tumors showed lower GLUT1 protein (*P* = 0.017), higher glutaminase 2 (GLS2) protein (*P* = 0.025) and higher GLS2 mRNA expression (*P* = 0.004), compared to EGFR wild‐type tumors. GLS mRNA expression was higher in *KRAS*‐mutated tumors (*P* = 0.019). *TP53*‐mutated tumors showed higher GLUT1 expression (*P* = 0.009).

**Conclusions:**

NSCLC is a heterogeneous disease, with differences in mutational status and metabolism‐related marker expression between adeno‐ and squamous cell NSCLCs, and also within adenocarcinoma subtypes. GLUT1 and SLC1A5 expression correlate with aggressive tumor behavior in adenocarcinomas but not in squamous cell NSCLCs. Therefore, these markers could steer treatment modification for subgroups of adenocarcinoma patients. *TP53, EGFR* and *KRAS* mutations are associated with expression of glucose and glutamine metabolism‐related markers in NSCLC.

## Key points

- Mutational status differs among adeno‐ (AC) and squamous cell (SCC) NSCLC.
- Metabolic markers differ between AC and SCC NSCLC, and also within AC subtypes.
- GLUT1 and SLC1A5 expression correlate with aggressive tumor behavior only in AC.
- These markers could select AC patients requiring treatment intensification.
- *TP53, EGFR* and *KRAS* mutations are associated with metabolic markers in NSCLC.

## Introduction

Non‐small cell lung cancer (NSCLC) is a heterogeneous disease regarding clinical, radiological, pathological, and molecular aspects.^1^, ^2^ In 2015, an update of the World Health Organization (WHO) classification was introduced, describing the major subtypes of adenocarcinomas demonstrating a predominantly lepidic, acinar, papillary, micropapillary, or solid growth pattern.^3^ This classification system for lung adenocarcinoma is an independent predictor of survival.^4^ Predominantly micropapillary and solid adenocarcinomas often have a more advanced TNM‐stage at diagnosis, a higher recurrence rate, and a worse survival relative to predominantly lepidic, acinar and papillary subtypes.^4^ Therefore, predominantly micropapillary and solid adenocarciomas have more aggressive behavior with early distant metastasis.

Reprogrammed tumor metabolism is one of the hallmarks of cancer.^5^, ^6^ To proliferate, a cell needs to replicate all of its cellular contents including DNA and organelles, and the cell has to sustain its redox status.^7^ Besides ATP, this requires reduced nicotinamide adenine dinucleotide phosphate (NADPH) and glutathione, and the biosynthesis of macromolecules essential for constructing a new cell.^8^ The main nutrients required for bioenergetics and macromolecular synthesis and thus tumor cell proliferation are glucose and glutamine. Increased glucose uptake by tumors to support enhanced glycolysis is accomplished by upregulation of glucose transporters (GLUTs).^9^ The higher glycolytic rate results in the production of lactate and acid, which are transported out of the cell by monocarboxylate transporters (MCTs) and carbonic anhydrase IX (CAIX).^6^ Important transporters and enzymes for glutamine uptake and metabolism are solute‐linked carrier family A1 member 5 (SLC1A5) and mitochondrial glutaminase (GLS), respectively.^10^, ^11^ GLS is the key enzyme in the conversion of glutamine to glutamate. GLS has two isoforms: GLS1 (kidney glutaminase) and GLS2 (liver glutaminase). GLS1 has two splice variants: kidney‐type glutaminase (KGA), and glutaminase C (GAC).^12^ This metabolic transformation is the result of complex interactions between a hypoxic tumor microenvironment and oncogenic mutations, such as epidermal growth factor receptor (*EGFR*), *TP53* and Kirsten rat sarcoma viral oncogene (*KRAS*).^9^, ^13^, ^14^, ^15^, ^16^ Higher glucose utilization correlates with aggressive tumor behavior and treatment resistance including radiotherapy.^6^ Therefore, tumor metabolism might be exploited in future treatment strategies.

Our previous analyses showed differences in GLUT1, CAIX, and MCT1 expression, vascular density and 18‐fluoro‐2‐deoxyglucose (^18^F‐FDG) uptake between adeno‐ and squamous cell NSCLCs, supporting differences in glucose tumor metabolism among NSCLC histological subtypes.^17^, ^18^, ^19^ In view of the association between glucose metabolism and aggressive tumor behavior, and the worse prognosis in predominantly micropapillary and solid adenocarcinomas, we further explored glucose and glutamine metabolism‐related markers in this study within squamous cell NSCLC and subclassifications of adenocarcinomas. Furthermore, expression of metabolism‐related markers was correlated with mutational status to examine oncogenic mutations that are associated with reprogrammed tumor metabolism in NSCLC.

## Methods

### Patients

**Figure 1:**
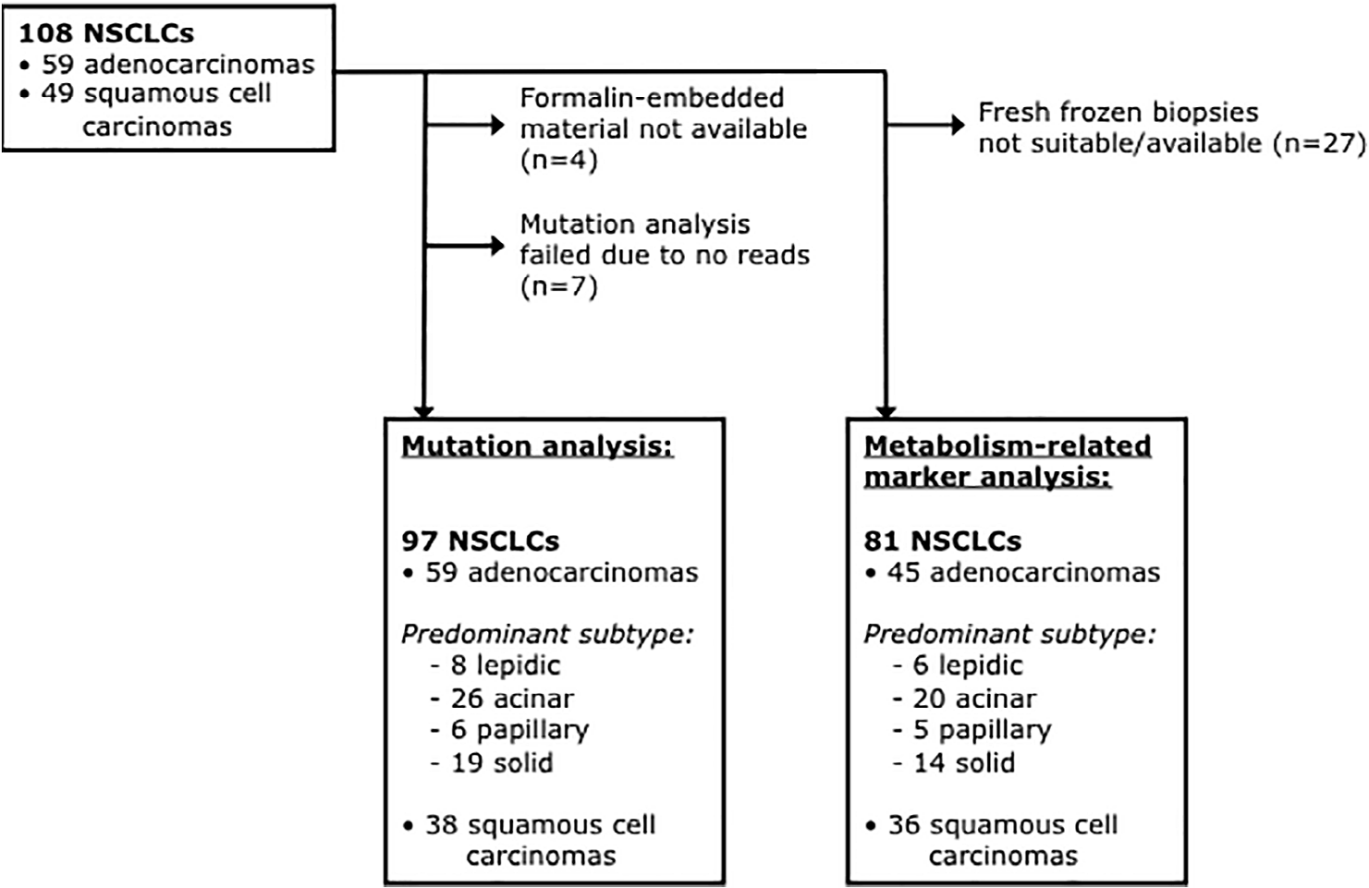
Flowchart of available NSCLC tissue for mutation and metabolism‐related marker analyses. NSCLC, non‐small cell lung carcinoma.

Patients who underwent a curative resection for stage I, II, and resectable stage IIIA, cN0‐1 adeno‐ or squamous cell NSCLC at the Radboud University Medical Center between January 2002 and December 2008 were included in this study as previously described.^18^ Of these 108 NSCLCs, fresh‐frozen lung resection biopsies for metabolism‐related marker analysis were available for 81 NSCLCs. Formalin‐embedded lung resection material for mutation analysis was available for 97 NSCLCs (Fig 1).

### Pathology

After surgery, the involved lung‐lobe was fixed in formaldehyde by inflating formaldehyde into the lobar/main bronchus and lobar/main artery. Fixated tumors were cut into transversal slices and maximum macroscopic transversal tumor dimension (mm) was then measured. In respect of a maximum tumor dimension in craniocaudal length, maximum tumor size (mm) was calculated by: slice thickness* (number of tumor positive slices minus 1). By subtracting 1, we took into account that the flanking slices mostly did not show full thickness tumor infiltration. Lung resection specimens were reviewed by a pathologist (ML) and typified on H&E stained slides according to the WHO 2015 classification.

### Immunofluorescent staining

Biopsies taken from fresh lung cancer resection specimens were snap frozen in liquid nitrogen and stored at −80°C until further processing. Sections of 5 μm, mounted on poly‐L‐lysine coated slides, were stored at −80°C until staining. Immunofluorescent staining of GLUT1, CAIX, MCT1, MCT4 and vascular density were as described previously by Meijer *et al*.^17^

Staining for SLC1A5 and GLS2 was performed on two consecutive tumor sections by incubating the sections with rabbit anti‐SLC1A5 (Abcam, Cambridge, UK) and rabbit anti‐GLS2 (ThermoFischer Scientific, Waltham, Massachusets, USA) respectively, diluted 1:150 in PAD (Bio‐Rad Laboratories Inc., Richmond, CA, USA), overnight at 4°C for SLC1A5 and 45 minutes at 37°C for GLS2. For SLC1A5, the second and third incubation took 30 minutes at 37°C with goat anti‐rabbitCy3 (Jackson Immuno Research Laboratories Inc.; West Grove, PA, USA) and donkey anti‐goatCy3 (Jackson) respectively, diluted 1:600 in PAD. For GLS2, the second incubation took 45 minutes at 37°C with goat anti‐rabbit Cy3, diluted 1:300 in PAD. After staining, sections were mounted in Fluoromount (SERVA Electrophoresis GmbH, Heidelberg, Germany).

### Fluorescence microscope image acquisition and analysis

Immunofluorescent image acquisition and analysis were as described by Meijer *et al*.^17^ In brief, slides were scanned at 100x magnification using a high‐resolution 12‐bit CCD camera (Coolsnap HQ, Roper Scientific Inc., Trenton, NJ, USA) on a fluorescence microscope (Axioskop, Zeiss, Göttingen, Germany). This resulted in grayscale images which were converted to binary images for further analysis. The tumor area was marked on the stained sections. Marker fractions were defined as the tumor area positive for the marker (binary images), divided by the total tumor area. Vascular density was calculated as the number of vascular structures per square millimeter.

For SLC1A5 and GLS2 staining, background intensity level hampered segmentation for binarizing the images. Therefore, these stainings required visual scoring. Fraction of tumor cells of the total tumor area stained for SLC1A5 and GLS2 were scored as <5%, 5%–20% and >20%. Slides were indivually scored by TM and JL. In case of disagreement, slides were discussed until agreement was reached.

### RT‐PCR

Total RNA was isolated with the '’s Total RNA purification kit (' Biotek Corp., Thorold, Ontario, Canada), and reversed transcribed using Gibco SuperScript II Reverse Transcriptase (Life technologies, Carlsbad, CA, USA). GLUT1, CAIX, MCT1 and MCT4 qPCR was described before.^18^ qPCR was performed with specific primers for SLC1A5 (FW: 5′‐ GAGCTGCTTATCCGCTTCTTC‐3′, RV: 5′‐ GGGGCGTACCACATGATCC‐3′), GLS (FW: 5′‐AGGGTCTGTTACCTAGCTTGG‐3′, RV: 5′‐ ACGTTCGCAATCCTGTAGATTT‐3′), GAC (FW: 5′‐ GGTCTCCTCCTCTGGATAAGATGG‐3′, RV: 5′‐ GATGTCCTCATTTGACTCAGGTGAC‐3′) and GLS2 (FW: 5′‐ GCCTGGGTGATTTGCTCTTTT‐3′, RV: 5′‐ CCTTTAGTGCAGTGGTGAACTT‐3′) on a CFX96 real‐time PCR detection system (Bio‐Rad Laboratories Inc) using SYBR Green. Levels are expressed as ratios of hypoxanthine ribosyltransferase (HPRT).^20^

### DNA isolation and mutation analysis

DNA isolation was essentially performed and samples sequenced using the smMIP gene panel as described by Eijkelenboom *et al*.^21^ Library preparation was performed as described by Neveling *et al*.^22^ See Data S1 for a detailed description of DNA isolation and mutation analysis.

### Statistical analysis

Statistical analyses were performed using SPSS 22.0 statistical software (SPSS Inc., Chicago, IL, USA). The Pearson Chi‐Square test and Spearman correlation were used to determine associations between clinicopathological characteristics, mutation status and metabolism‐related markers on a trichotomous scale. The correlation coefficient (r) between metabolism‐related markers on a continuous scale and clinicopathological parameters was calculated using the Pearson's and Spearman's Rank test where appropriate (testing for Gaussian distribution by one‐sample Kolmogorov‐Smirnov Test). Differences in metabolism‐related marker expression between histological subtypes or mutated versus wild‐type tumors were measured with the Mann‐Whitney U test or the Independent‐samples *t*‐test where appropriate. A *P* < 0.05 was considered statistically significant.

## Results

### Glucose and glutamine metabolism‐related markers in NSCLC histological subtypes

**Figure 2:**
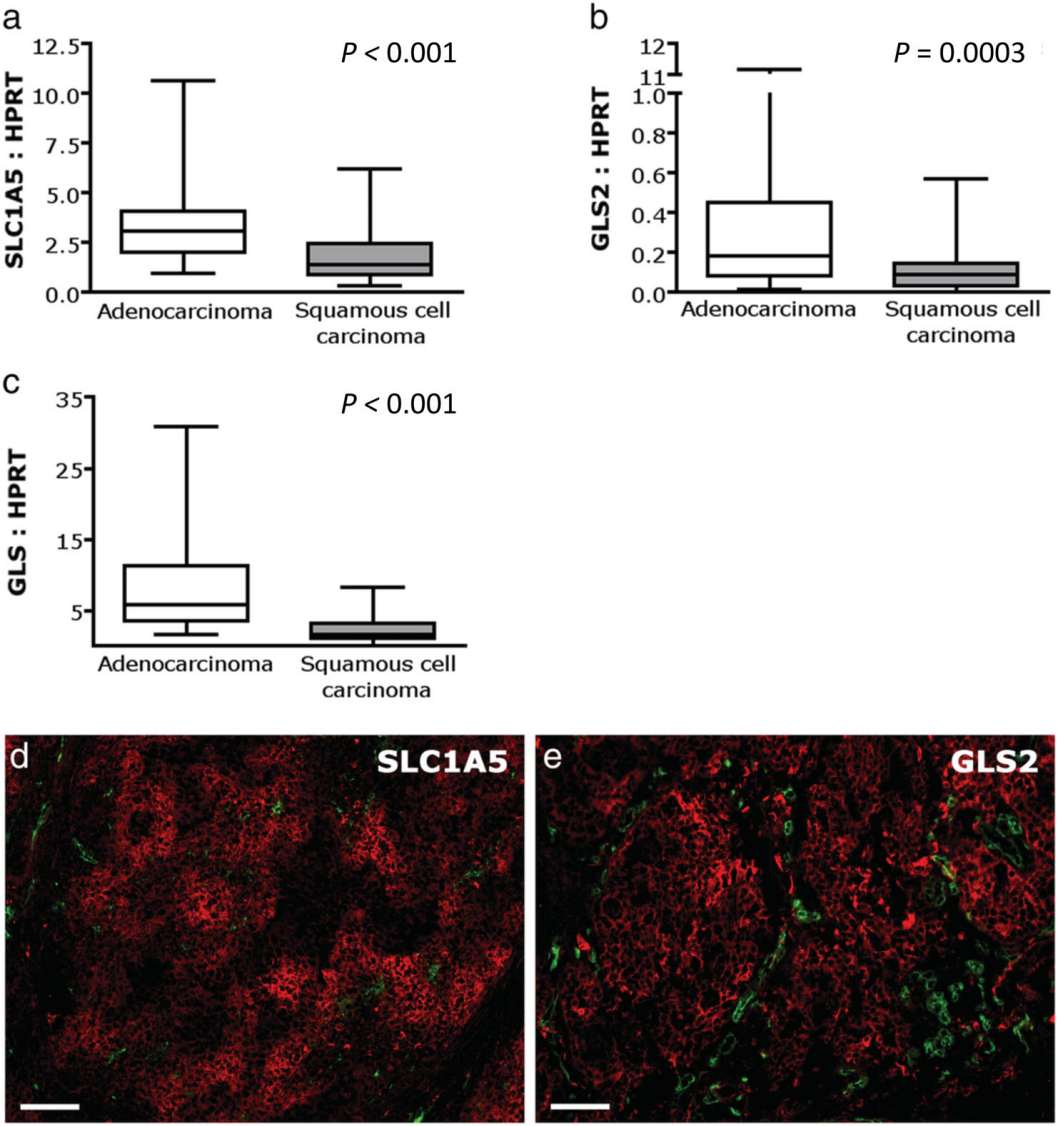
Glutamine transporter and glutaminase mRNA expression in adeno‐ versus squamous cell non‐small cell lung cancer. Expression of glutamine transporter SLC1A5 mRNA (**a**), glutaminase mRNA and (**b**) glutaminase 2 mRNA (**c**) is higher in adenocarcinomas relative to squamous cell carcinomas. Levels are expressed as ratios of HPRT. (**d**,**e**) Immunofluorescent images of adeno NSCLCs showing SLC1A5 (**d**) and GLS2 (**e**) expression. Red, SLC1A5 or GLS2; green, vessels; magnification 100x; scale bars represent 100 μm. GLS, glutaminase; SLC1A5, solute‐linked carrier family A1 member 5.

**Figure 3:**
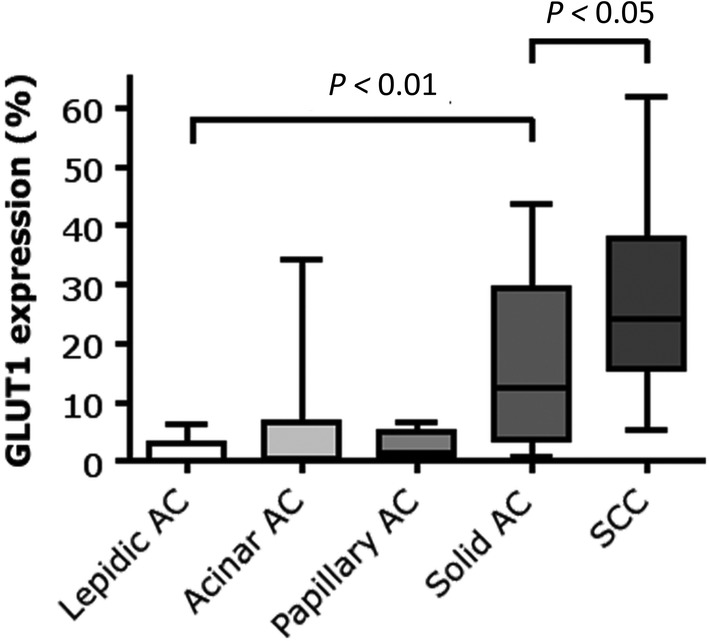
GLUT1 expression within NSCLC histological subtypes classified according to the WHO 2015 subclassification. Within adenocarcinomas, GLUT1 expression is highest in solid adenocarcinomas. Squamous cell carcinomas demonstrate an even higher expression of GLUT1 relative to solid adenocarcinomas. GLUT1 fractions are based on immunofluorescent stainings and are defined as the tumor area positive for the marker, divided by the total tumor area. AC, adenocarcinoma; GLUT1, glucose transporter 1; SCC, squamous cell carcinoma.

Adenocarcinomas showed higher expression of SLC1A5 and GLS2 in both protein (*P* = 0.08 and *P* = 0.019, respectively) and mRNA level (*P* < 0.001 and *P* = 0.003, respectively) (Fig 2, Table 1), compared to squamous cell carcinomas. Also, GLS mRNA level was higher in adenocarcinomas than squamous cell carcinomas (*P* < 0.001) (Fig 2c). In adenocarcinomas, SLC1A5 mRNA expression correlated with tumor size (r(p) = 0.41, *P* = 0.005). Furthermore, SLC1A5 protein expression was significantly higher in adenocarcinomas with lymph node metastases compared to node negative tumors (r(s) = 0.34, *P* = 0.028) and in adenocarcinomas with higher pTNM stage (r(s) = 0.39, *P* = 0.009) (Table 2). Glutamine metabolism‐related markers and vascular density were not different within adenocarcinoma subclassifications. GLUT1 expression differed among adenocarcinoma subclassifications (*P* = 0.002), and was significantly higher in predominantly solid compared to predominantly lepidic adenocarcinomas (*P* < 0.01) (Fig 3). Squamous cell carcinomas had an even higher GLUT1 expression compared to predominantly solid adenocarcinomas: median GLUT1 expression was 24.1% (range 5.5–62) versus a median expression of 12.6% (range 0.8–43.8) (*P* < 0.05) (Fig 3).

**Table 1:** Glutamine metabolism‐related markers in relation to clinicopathological characteristics and *EGFR* mutation status. Glutamine transporter SLC1A5 and glutaminase GLS2 protein expression in adeno‐ versus squamous cell non‐small cell lung cancer

	Adenocarcinoma	Squamous cell carcinoma	*P*‐value
**SLC1A5**			
<5%	19 (44.2%)	21 (60%)	*P* = 0.08
5–20%	11 (25.6%)	10 (28.6%)	
>20%	13 (30.2%)	4 (11.4%)	
**GLS2**			
<5%	29 (67.4%)	31 (88.6%)	*P* = 0.019
5–20%	5 (11.6%)	3 (8.3%)	
>20%	9 (20.9%)	1 (2.8%)	

Two tissue samples were missing for adenocarcinomas and one sample was missing for squamous cell carcinomas, resulting in 43 adenocarcinomas and 35 squamous cell carcinomas for glutamine metabolism‐related marker analysis.

GLS2, glutaminase 2; SLC1A5, solute carrier family 1 (neutral amino acid transporter) member 5.

**Table 2:** Glutamine metabolism‐related markers in relation to clinicopathological characteristics and *EGFR* mutation status. Glutamine transporter SLC1A5 protein expression is higher in adenocarcinomas with a worse pTNM stage

	SLC1A5			
	<5%	5–20%	>20%	*P*‐value
**pTNM stage**				
Stage I	17 (65.4%)	2 (7.7%)	7 (26.9%)	*P* = 0.009
Stage II	1 (11.1%)	7 (77.8%)	1 (11.1%)	
Stage III	0 (0%)	2 (28.6%)	5 (71.4%)	
Stage IV	1 (100%)	0 (0%)	0 (0%)	

Two tissue samples were missing for adenocarcinomas, resulting in 43 adenocarcinomas.

SLC1A5, Solute carrier family 1 (neutral amino acid transporter) member 5.

### Mutation analysis

Mutations are described in Table 3 and Data S2. *TP53* and *PIK3CA* mutations were more prevalent in squamous cell carcinomas than in adenocarcinomas (97.4% vs. 71.2%, *P* = 0.001; 10.5% vs. 0%, *P* = 0.011 respectively). *EGFR* and *KRAS* mutations were more frequently observed in adenocarcinomas (10.2% vs. 0%, *P* = 0.042; 35.6% vs. 5.3%, *P* = 0.001 respectively). *EGFR* mutations were found in predominantly lepidic and acinar adenocarcinomas and not in adenocarcinomas with a predominantly papillary or solid growth pattern (not significant). Other mutations (*AKT1, BRAF, IDH1/2, KIT, PDGFRA*) were sporadically demonstrated and *ERBB2* and *JAK2* mutations were not observed.

**Table 3:** Mutation analysis in adeno‐ versus squamous cell non‐small cell lung cancer

	Adenocarcinomas (*n* = 59)				
	All	Lepidic	Acinar	Papillary	Solid
Mutation	(*n* = 59)	(*n* = 8)	(*n* = 26)	(*n* = 6)	(*n* = 19)
AKT1	0	0	0	0	0
BRAF	1 (1.7%)	0	0	0	1 (5.3%)
EGFR	6 (10.2%)	2 (25%)	4 (15.4%)	0	0
ERBB2	0	0	0	0	0
IDH1	0	0	0	0	0
IDH2	1 (1.7%)	0	0	0	1 (5.3%)
JAK2	0 (0%)	0	0	0	0
KIT	2 (4.3%)	0	2 (7.7%)	0	0
KRAS	21 (35.6%)	4 (50%)	10 (38.5%)	1 (17%)	6 (31.6%)
PDGFRA	1 (1.7%)	0	0	0	1 (5.3%)
PIK3CA	0	0	0	0	0
TP53	42 (71.2%)	5 (62.5%)	19 (73.1%)	3 (50%)	15 (78.9%)
**Mutation**	**Squamous cell**		***P*‐value**		
	**carcinoma (*n* = 38)**		**AC versus SCC**		
AKT1	1 (2.6%)				
BRAF	0				
EGFR	0		*P* = 0.042		
ERBB2	0				
IDH1	1 (2.6%)				
IDH2	1 (2.6%)				
JAK2	0				
KIT	1 (2.6%)				
KRAS	2 (5.3%)		*P* = 0.001		
PDGFRA	0				
PIK3CA	4 (10.5%)		*P* = 0.011		
TP53	37 (97.4%)		*P* = 0.001		

AKT, protein kinase B; BRAF, v‐raf murine sarcoma viral oncogene homolog B; EGFR, epidermal growth factor receptor; IDH, isocitrate dehydrogenase; KIT, KIT proto‐oncogene receptor tyrosine kinase; KRAS, Kirsten rat sarcoma viral oncogene; PDGFRA, platelet‐derived growth factor receptor A; PIK3CA, gene encoding for the phosphatidylinositol 3‐kinase (PI3K) catalytic subunit; TP53: transformation‐related protein 53.

### Association between metabolism‐related marker expression and mutational status

**Figure 4:**
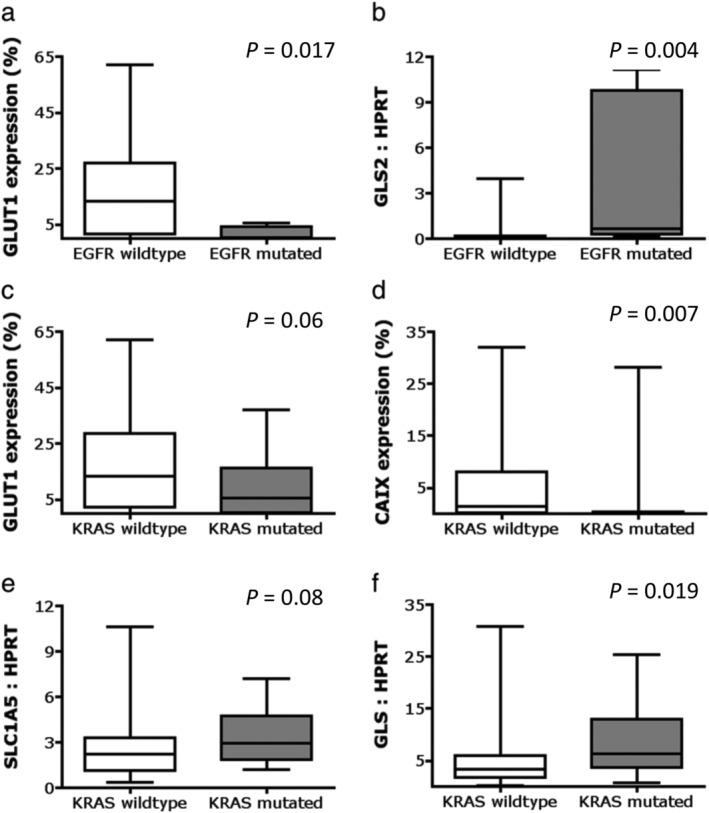
Association of metabolism‐related marker expression with mutation status in NSCLC. GLUT1 protein expression is higher in EGFR wild‐type than *EGFR*‐mutated NSCLCs. GLS2 mRNA expression is higher in *EGFR*‐mutated versus EGFR wild‐type NSCLCs. GLUT1 protein expression in KRAS wild‐type versus *KRAS*‐mutated NSCLCs. CAIX protein expression is higher in KRAS wild‐type versus *KRAS*‐mutated NSCLCs. SLC1A5 mRNA expression in KRAS wild‐type versus *KRAS*‐mutated NSCLCs. GLS mRNA expression is higher in *KRAS*‐mutated versus KRAS wild‐type NSCLCs. CAIX, carbonic anhydrase IX; EGFR, epidermal growth factor receptor; GLS, glutaminase; GLUT1, glucose transporter 1; KRAS, Kirsten rat sarcoma viral oncogene; SLC1A5, solute‐linked carrier family A1 member 5.

*EGFR*‐mutated tumors showed lower GLUT1 protein expression (*P* = 0.017; Fig 4a), corresponding with the fact that predominantly lepidic and acinar adenocarcinomas are *EGFR* mutated and demonstrate low GLUT1 expression. Furthermore, in *EGFR* mutated tumors, higher expression of GLS2 protein (*P* = 0.025; Table 4) and GLS2 mRNA was observed compared to EGFR wild‐type tumors (*P* = 0.004; Fig 4b).

**Table 4:** Glutamine metabolism‐related markers in relation to clinicopathological characteristics and *EGFR* mutation status. Glutaminase GLS2 protein expression in EGFR wild‐type versus *EGFR*‐mutated non‐small cell lung cancer

	EGFR wild‐type	*EGFR*‐mutated	*P*‐value
**GLS2**			
<5%	51 (78.5%)	2 (40%)	0.025
5–20%	7 (10.8%)	0 (0%)	
>20%	7 (10.8%)	3 (60%)	

EGFR, epidermal growth factor receptor; GLS2, glutaminase 2.

*KRAS* mutated tumors showed a trend toward lower GLUT1 protein expression (*P* = 0.06; Fig 4c), and demonstrated a significantly lower CAIX protein expression (*P* = 0.007; Fig 4d). *KRAS* mutation status correlated with higher glutamine metabolism, as observed by a trend toward higher SLC1A5 mRNA expression (*P* = 0.08; Fig 4e) and a significantly higher GLS mRNA expression (*P* = 0.019; Fig 4f).

*TP53* mutation status correlated significantly with higher GLUT1 expression (*P* = 0.009). Median GLUT1 expression was 0.3% (range 0%–43.8%) for *TP53* wild‐type tumors versus 13.3% (range 0%–62%) for *TP53* mutated tumors. Frequencies of other mutations were too low for correlation analysis with metabolism‐related markers.

## Discussion

Differences in metabolism between NSCLC histologies may have consequences for ^18^F‐FDG positron emission tomography (PET) interpretation and prognosis in the clinic after surgery and for radiotherapy efficacy.^6^, ^18^ Here, we describe differences in mutational status and metabolism‐related marker expression between adeno‐ and squamous cell NSCLCs.

### NSCLC tumor cell metabolism in relation to histological subtype and aggressive tumor behavior

In an earlier study we described the correlation between high GLUT1 expression with poor differentiation grade and lymph node metastasis at diagnosis in adenocarcinomas in this same cohort of NSCLC patients.^17^ Also, we found a worse survival in adenocarcinomas with high GLUT1 and high MCT4 expression or high total lesion glycolysis (TLG) on ^18^F‐FDG‐PET/CT.^17^, ^18^ In this study, we further explored glucose metabolism‐related markers in adenocarcinoma subtypes. We showed that the adenocarcinoma subtype with the most aggressive tumor behavior, for example predominantly solid adenocarcinoma, has the highest GLUT1 expression. This is in agreement with the fact that adenocarcinomas with a predominantly micropapillary or solid growth pattern exhibit higher maximum standardized uptake value (SUV_max_) on ^18^F‐FDG‐PET than predominantly acinar or papillary tumors. Predominantly lepidic adenocarcinomas demonstrate the lowest FDG uptake.^23^, ^24^

In our study, several markers related to glutamine transport and metabolism, on both protein and mRNA level, were significantly higher in adeno‐ than squamous cell NSCLCs. This is in contrast to the findings of others, who demonstrated that SLC1A5 expression is associated with squamous cell histology.^10^, ^25^, ^26^ In an in vivo mice study, the glutamine tracer 5‐^11^C‐(2S)‐glutamine (^11^C‐Gln) uptake was higher in a squamous cell carcinoma than an adenocarcinoma, although *n* = 1 for both histologies, so the question is whether these tumors are a reliable representation of these histologies.^27^ However, we also examined GLS2 protein expression and mRNA expression of SLC1A5, GLS and GLS2. All our findings indicate a higher glutamine consumption and metabolism in adenocarcinomas relative to squamous cell carcinomas. It would be of interest to study the uptake of glutamine PET tracers in NSCLC patients to validate the mRNA and protein expression profiles, and to get more insight in glutamine metabolism of adeno‐ versus squamous cell NSCLC.

Our study revealed that SLC1A5 protein expression was significantly higher in adenocarcinomas with lymph node metastases at diagnosis compared to node negative tumors, indicating more aggressive tumor behavior upon higher SLC1A5 expression. In a Japanese study of adenocarcinomas, SLC1A5 expression also correlated with advanced tumor stage and lymph node metastasis.^28^ Furthermore, a multivariate analysis demonstrated that SLC1A5 expression was an independent marker of poor overall survival for adenocarcinoma patients, while this was not observed for squamous cell NSCLCs.^28^ Other studies showed that SLC1A5 overexpression in the primary tumor or lymph node metastasis was found to be an independent prognostic factor for overall survival for NSCLC.^25^, ^26^ However, these studies did not distinguish adeno‐ versus squamous cell NSCLC.

High expression of LAT1 (L amino acid transporter) or SLC1A5, but especially coexpression of these transporters, correlated with worse overall survival in lung adenocarcinomas.^29^ Therefore, besides a higher glycolytic rate, also a higher rate of glutamine metabolism correlates with biologically aggressive tumor behavior and worse survival in lung adenocarcinomas. This indicates that these metabolic markers could select a group of lung adenocarcinoma patients requiring intensification of systemic treatment in the adjuvant setting after surgery, for example by inhibition of tumor metabolism. However, to validate these results of the prognostic potential of metabolism‐related markers in relation to histology and to define cutoff points guiding intensification of systemic treatment, a larger patient cohort should be investigated in a prospective observational study. These results regarding differences in glucose metabolism may also apply for other tumor entities. Adenocarcinomas of the cervix and esophagus have a higher potential to metastasize relative to squamous cell carcinomas, and metabolic tumor volume on ^18^F‐FDG PET is of prognostic value only for esophageal adenocarcinoma.^30^, ^31^, ^32^

### Oncogenic mutations that drive tumor metabolism in NSCLC

Wild‐type p53 inhibits glycolysis by repressing glucose transporters and upregulating expression of TP53‐induced glycolysis and apoptosis regulator (TIGAR). Wild‐type p53 also supports expression of the tumor suppressor phosphatase and tensin homolog (PTEN), which inhibits AKT activity.^7^, ^9^, ^33^ Thus, glycolysis can be promoted by loss of p53 function. However, the stimulating effect on glycolysis varies among H1299 NSCLC cells with different mutational p53 proteins. In two of nine p53 mutants, no increase in glycolysis was observed. Therefore, mutant p53 proteins can have differential phenotypic impact on metabolic pathways.^34^ Overall, the link between *TP53* mutation and glycolysis is in agreement with our result showing higher GLUT1 expression in *TP53*‐mutated tumors.

Frequency of two other mutations rewiring tumor metabolism, ie. *EGFR* and *KRAS* mutations, are much higher in adeno‐ than squamous cell carcinomas. In non‐Asian cohorts, *EGFR* mutation frequencies of 10%–30% are described in lung adenocarcinomas. *KRAS* mutations are found in 22–34% of adenocarcinomas, consistent with our observation.^35^ The oncogene *KRAS* activates the RAF/MEK/ERK pathway, leading to upregulation of MYC transcription factor.^36^ *RAS* promotes glucose uptake, glycolytic flux and channeling of intermediates into the pentose phosphate pathway through upregulation of GLUT1 and hexokinase among others.^9^, ^37^, ^38^ Furthermore, glutamine is the major carbon source for the tricarboxylic acid cycle when RAS is activated.^38^ SLC1A5 is upregulated through KRAS signaling in colorectal cancer,^39^ and the conversion of glutamine to glutamate by GLS is modulated by RAS.^38^ This is in agreement with our findings that *KRAS* mutated tumors show a trend toward a higher SLC1A5 mRNA expression and a significantly higher GLS mRNA expression. EGFR affects tumor metabolism through activation of the PI3K/AKT/mTOR signaling pathway.^40^ In our study, *EGFR* mutated tumors had a higher expression of GLS2 protein and GLS2 mRNA compared to EGFR wild‐type tumors. The EGFR tyrosine kinase inhibitor (EGFR‐TKI) erlotinib reduces expression of glutamine transporters (SLC1A5 and SLC38A1) and GLS, and declines glutamine uptake.^41^

These results support the linkage between oncogenicmutated *KRAS* and *EGFR* signaling and aerobic glycolysis and glutamine metabolism in adenocarcinomas,^38^, ^40^, ^42^ and are in agreement with tumor microenvironmental characteristics: adenocarcinomas show better vascularization relative to squamous cell carcinomas, GLUT1 and MCT4 expression in a nonhypoxia‐related pattern, and a lower rate constant of cytoplasmic phosphorylation of ^18^F‐FDG and higher blood volume fraction on dynamic ^18^F‐FDG‐PET.^17^, ^18^, ^19^ However, a limitation is the descriptive character of this study. These associations between mutations and expression of metabolism‐related markers do not necessarily imply that these mutations regulate metabolism of lung adenocarcinoma. Another limitation of this study is the question whether a single biopsy is representative for the whole tumor. Genetic and environmental parameters influence the phenotype and thus cell metabolism resulting in metabolic heterogeneity. Hypoxia and tumor acidosis fluctuate with time and may influence metabolism in specific tumor areas at a given time.^43^ Metabolic heterogeneity is identified between but also within lung tumors.^44^

Squamous cell carcinomas are phenotypically characterized by a hypoxic tumor microenvironment,^17^, ^18^, ^19^ a microenvironmental characteristic associated with radioresistance.^45^ Squamous cell NSCLCs have a poor vascularization, more often show necrosis, demonstrate GLUT1 and MCT4 expression in a hypoxia‐related pattern, and show a higher rate constant of cytoplasmic phosphorylation of ^18^F‐FDG and lower blood volume fraction on dynamic ^18^F‐FDG‐PET.^17^, ^18^, ^19^ This hypoxic tumor microenvironment activates the hypoxia‐inducible factor 1 (HIF‐1) pathway. HIF‐1 initiates transcription of genes that encode transporters and enzymes regulating glycolysis and the pentose phosphate pathway.^6^ However, besides hypoxia, squamous cell carcinomas exhibit low frequencies of *PIK3CA, AKT1* and *PTEN* mutations. In our study population, *AKT1* and *PIK3CA* mutations were only found in squamous cell carcinomas, with frequencies of 2.6% and 10.5%, respectively. This is in agreement with the literature, describing a higher frequency of *PIK3CA* mutations in squamous cell carcinomas (7.1–8.9%) than adenocarcinomas (0.9–2.9%).^46^, ^47^ Also, *PTEN* mutations are more frequently observed in squamous cell carcinomas (10.7%) than adenocarcinomas (1.7%).^47^ The prevalence of *AKT1* mutation is low, but related to squamous cell histology.^48^ These mutations could regulate metabolism through PI3K/AKT signaling in a few squamous cell NSCLC. However, frequencies were too low to perform correlation analysis with metabolism‐related markers. In our study, only gain‐ and loss‐of‐function mutations were assessed, but other genetic alterations, such as amplifications, were not tested. PIK3CA gene amplification is found in 46% of squamous cell carcinomas, with a correlation between PIK3CA genomic copy number and PIK3CA and GLUT1 mRNA expression.^49^ Squamous cell carcinomas exhibit increased AKT signaling activities compared to adenocarcinomas in terms of higher expression of p‐AKT, and the downstream targets p‐4EBP1 and p‐S6, which promote HIF‐1α mRNA translation.^49^, ^50^ AKT is activated by hypoxia and this pathway is relevant for cell survival of hypoxia. Besides the HIF‐1 pathway, PI3K/AKT signaling induced by hypoxia seems to be important for hypoxic cell survival and regulation of metabolism in squamous cell carcinomas.^50^

The inverse relationship between *KRAS* and *EGFR* mutation with GLUT1 expression seems to contradict the regulatory effect of these mutations on glycolysis. However, this finding might be biased by the fact that hypoxia is likely to be a very strong regulator of glycolysis in squamous cell carcinomas, in which *KRAS* and *EGFR* mutations are not (often) found. This inverse relationship is in agreement with the finding that SUV_max_ on ^18^F‐FDG‐PET is significantly lower in *KRAS* or *EGFR* mutated NSCLCs.^51^

In conclusion, NSCLC is a heterogeneous disease with differences in mutational status and expression of metabolism‐related markers between adeno‐ and squamous cell NSCLCs, but also within adenocarcinoma subtypes. GLUT1 and SLC1A5 expression correlate with aggressive tumor behavior in adenocarcinomas, but not in squamous cell NSCLCs. This indicates that these markers could select a group of adenocarcinoma patients requiring intensification of treatment. Regulation of tumor metabolism is complex. Associations found in this study suggest that *EGFR, KRAS* and *TP53* mutations might drive tumor metabolism in adenocarcinomas. In squamous cell carcinomas, *TP53* mutations and hypoxia activating the HIF‐1 pathway and PI3K/AKT signaling are associated with glucose metabolism‐related markers and could be the main drivers of metabolism in this histological subtype.

## Disclosure

The authors declare no potential conflicts of interest.

## Supporting information


**Data S1** DNA isolation.

**Data S2** Supplementary File.
