# Supplementary material for: Glucose and glutamine metabolism in relation to mutational status in NSCLC histological subtypes
Source: Thorac Cancer. 2019 Oct 30;10(12):2289–99. doi: 10.1111/1759-7714.13226 (PMC6885430; doi:10.1111/1759-7714.13226)
Supplement: Supplementary file 1 — Data S1 DNA isolation. [file TCA-10-2289-s001.docx]

**Supplementary data 1**

**DNA isolation**

3 × 20 μm sections were digested at 56°C for 1 hour in the presence of TET-lysis buffer (10 mmol/L Tris/HCl pH8.5, 1 mmol/L EDTA pH8.0, 0.01% Tween-20) with 5% Chelex-100 (143 to 2832; Bio-Rad, Hercules, CA), 15 μg/mL GlycoBlue (AM9516; Thermo Fisher, Waltham, MA), and 400 μg proteinase K (19133; Qiagen, Valencia, CA), followed by inactivation at 95°C for 10 minutes. The supernatant was cooled on ice and precipitated in the presence of 70% EtOH and 1/10 volume 3M NaAc (pH 5.2). Pellets were washed with cold 70% EtOH and dissolved in 80 μL Tris-EDTA, and DNA concentration was determined using the Qubit Broad Range Kit (Q32853; Thermo Fisher).

**Mutational analysis**

100 ng (in a maximum volume of 7 μl) of genomic DNA was used as input in a 20-μL volume with a total capture volume of 25 μL, including the (diluted) phosphorylated smMIP pool (smMIP:gDNA ratio of 3200:1), 1 unit of Ampligase DNA ligase (A0110K; EpiBio, Madison, WI) with Ampligase Buffer (A1905B, DNA ligase buffer), 3.2 units of Hemo Klentaq (M0332; New England Biolabs), and 8 μmol of dNTPs (28-4065-20/-12/-22/-32; GE Healthcare, Little Chalfont, UK). After denaturation (95°C for 10 minutes) the mix was incubated for probe hybridization, extension, and ligation at 60°C for 18 hours and cooled before exonuclease treatment. Exonuclease I (10 units; M0293; New England Biolabs) and III (50 units; M0206; New England Biolabs) and Ampligase Buffer (see above) were added to the capture volume, adding up to a total of 27 μL, and incubated for 45 minutes at 37°C, followed by inactivation at 95°C for 2 minutes. A total of 10 μL of the exonuclease-treated capture was used for PCR in a total volume of 25 μL with 25 nmol common forward primer and barcoded reverse primers13 and iProof high-fidelity master mix (1725310; Bio-Rad). The resulting PCR products were pooled before purification with 0.8× volume of Agencourt Ampure XP Beads (A63881; Beckman Coulter, Brea, CA). Hybridization reactions were pipetted using a Microlab STARplus robot (Hamilton, Reno, NV). The exonuclease treatment was performed manually to prevent exonuclease contamination in the pre- and post-PCR environments. The post-hybridization PCR was performed using a MicroLab STARlet Replicator Robot (Hamilton). All samples were subsequently pooled on a MicroLab Starlet Replicator Robot (Hamilton).

The purified libraries were denatured and diluted to a concentration of 1.2 pmol/L. Sequencing was performed on a NextSeq500 instrument (Illumina, San Diego, CA) according to the manufacturer's protocol (300 cycles Mid Output sequencing kit, v2), resulting in 2 × 150 bp paired-end reads.

Fastq files were analyzed using commercial analysis software (Sequence Pilot version 4.2.0; JSI medical systems, Ettenheim, Germany). The following settings were used for single-molecule–directed consensus: Tags active, yes; R1 tag length, 8; R2 tag length, 0; Min abs. cov. cons., 1; Min per. cov. cons., 50%; Ignore cons. read thresh., 30; Ignore N tags, yes, and Ignore low Qs tags, yes. Proper identification of sequence variants greatly relies on the variables for variant calling, such as the minimal number of mutant unique smMIPs (>2 in our settings) and the minimal mutant allele frequency (≥1%). The following settings were used for variant calling using Sequence Pilot: Required Coverage/Min abs. cov., 40 combined; Mutations/Min abs. cov., 5 combined; Min % cov., 1% per dir. PIK3CA pseudogene reads were removed from the alignment and subsequent analysis. After variant calling using the commercial software, all variants were manually inspected and curated.
